# Radiographic parameter-driven decision tree reliably predicts aseptic mechanical failure of compressive osseointegration fixation

**DOI:** 10.1080/17453674.2020.1716295

**Published:** 2020-01-21

**Authors:** Ryland Kagan, Lindsay Parlee, Brooke Beckett, James B Hayden, Kenneth R Gundle, Yee-Cheen Doung

**Affiliations:** a Department of Orthopaedics and Rehabilitation, Oregon Health & Science University, Portland, OR;; b Department of Diagnostic Radiology, Oregon Health & Science University, Portland, OR;; c Operative Care Division, Portland Veterans Administration Medical Center, Portland, OR, USA

## Abstract

Background and purpose — Compressive osseointegration fixation is an alternative to intramedullary fixation for endoprosthetic reconstruction. Mechanical failure of compressive osseointegration presents differently on radiographs than stemmed implants, therefore we aimed to develop a reliable radiographic method to determine stable integration.

Patients and methods — 8 reviewers evaluated 11 radiographic parameters from 29 patients twice, 2 months apart. Interclass correlation coefficients (ICCs) were used to assess test–retest and inter-rater reliability. We constructed a fast and frugal decision tree using radiographic parameters with substantial test–retest agreement, and then tested using radiographs from a new cohort of 49 patients. The model’s predictions were compared with clinical outcomes and a confusion matrix was generated.

Results — 6 of 8 reviewers had non-significant intra-rater ICCs for ≥ one parameter; all inter-rater ICCs were highly reliable (p < 0.001). Change in length between the top of the spindle sleeve and bottom of the anchor plug (ICC 0.98), bone cortex hypertrophy (ICC 0.86), and bone pin hypertrophy (ICC 0.81) were used to create the decision tree. The sensitivity and specificity of the training cohort were 100% (95% CI 52–100) and 87% (CI 74–94) respectively. The decision tree demonstrated 100% (CI 40–100) sensitivity and 89% (CI 75–96) specificity with the test cohort.

Interpretation — A stable spindle length and at least 3 cortices with bone hypertrophy at the implant interface predicts stable osseointegration; failure is predicted in the absence of bone hypertrophy at the implant interface if the pin sites show hypertrophy. Thus, our decision tree can guide clinicians as they follow patients with compressive osseo­integration implants.

Compressive osseointegration fixation offers an alternative to cemented or non-cemented intramedullary stems for endoprosthetic reconstruction. This technology creates a stable, high-pressure bone–implant interface that theoretically avoids stress shielding (Frost 1994, Kramer et al. 2008, Bini et al. 2000). The continuous force at the bone–implant interface creates ingrowth into the porous surface of the component, resulting in stable integration. A spring system within the Compress (Zimmer Biomet, Warsaw, IN, USA) immediately applies high compressive forces to the bone–implant interface (Figure 1). This technology has the potential to decrease the rate of aseptic mechanical failure (loosening), allow for stable short-segment fixation, and preserve bone stock if revision is required (Bini et al. 2000, Calvert et al. 2014, Monument et al. 2015).

**Figure 1.**
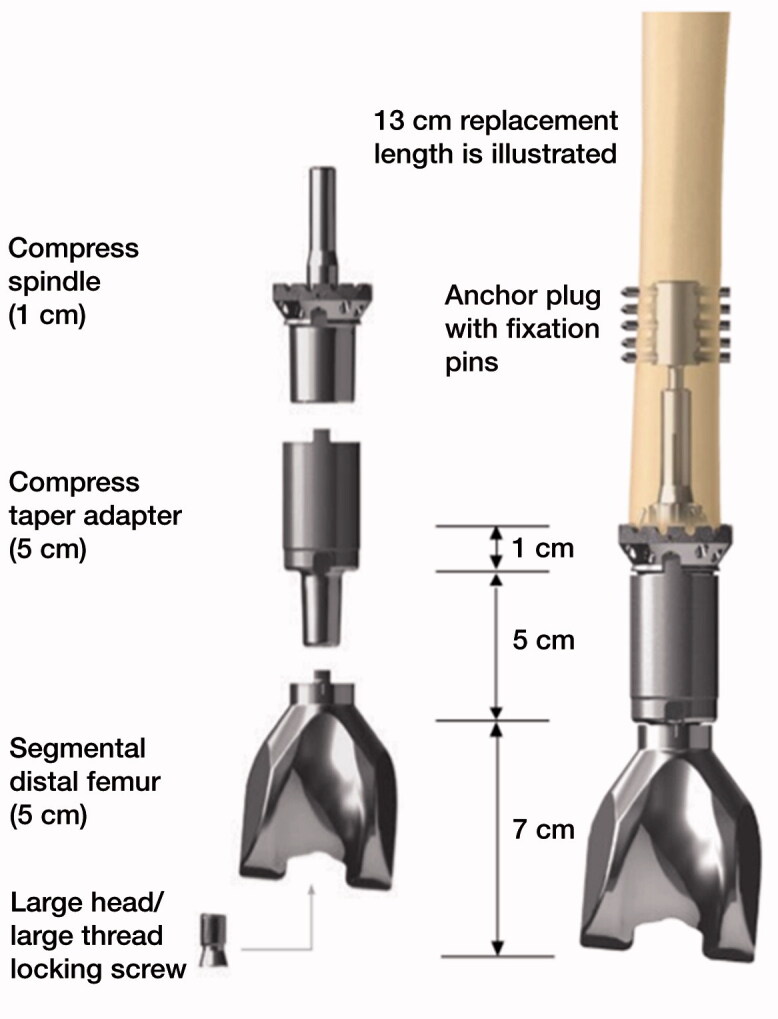
Compress components with distal femoral adaptor.

Loosening of compressive osseointegration technology is thought to occur sooner than with traditional intramedullary stems, which fail later due to stress shielding (Pedtke et al. 2012, Kagan et al. 2017). Due to these differences, radiographic parameters used to assess other forms of endoprosthetic reconstruction may be insufficient for identifying and predicting aseptic mechanical failure of compressive osseointegration fixation.

Bone hypertrophy visible at the bone–implant interface is thought to represent stable integration and thus it is a radiographic parameter often used to evaluate compressive osseointegration technology (Kramer et al. 2008, Pedtke et al. 2012, Healey et al. 2013, Zimel et al. 2016). Radiographic findings indicative of loosening include progressive gross decrease in the spindle height (Pedtke et al. 2012), deformation of the implant that suggests bending or breaking of the device (Healey et al. 2013), and bony atrophy at the bone–implant interface (Healey et al. 2013. However, there is no suitable methodology for evaluating aseptic mechanical failure of this technology (Pedtke et al. 2012, Healey et al. 2013).

Lacking validated gold standards, quantitative radiographically based tools such as the Radiographic Union Score for Tibial (RUST) fractures (Whelan et al. 2010) or the Radiographic Union Score for Hip (RUSH) score (Chiavaras et al. 2013) have been developed. These tools show how systematically evaluating radiographic parameters improves reliability and reproducibility. Fast and frugal decision trees (FFTs) are a classification heuristic that provides dichotomous choices in series (Phillips et al. 2017). This model has been used to stratify patient risk for acute myocardial injury (Green and Mehr 1997). If reliable radiographic parameters suggestive of compressive osseointegrative failure are used as decision points, an FFT may be a suitable model for categorizing patient risk of aseptic mechanical failure.

To evaluate whether there has been stable integration of compressive osseointegration technology we asked: What is a reliable radiographic method for determining stable bone–implant fixation? What radiographic parameters best show failure of fixation? Can an FFT be used to classify radiographs into stable and failed fixation categories?

## Patients and methods

In this 2-phase cohort study, separate cohorts of patient radiographs were evaluated to develop and validate (i.e., train and test) a model to predict aseptic mechanical failure of the Compress in the lower extremity.

### Training cohort

Radiographs from patients who received Compress implants between 2006 and 2014 were reviewed. During this time, surgeons at one center implanted 132 lower extremity Compress devices in 109 patients. Indications for compressive osseointegration fixation use included reconstruction of the proximal femur, distal femur, and proximal tibia where there was massive bone loss necessitating endoprosthetic reconstruction. Patients were considered for this technology if they had previous failed arthroplasty, fracture nonunion, malunion, or required a reconstruction after an oncologic resection. Older age was not a contraindication for use. Study inclusion criteria were a minimum clinical and radiographic follow-up of 2 years.

The compressive force used on each patient was determined based on the cortical thickness of the bone. The spindle size and shape were determined, based on the individual patient anatomy, at the time of surgery by 1 of the 2 senior authors (YCD, JBH). The diameter chosen was always larger than the largest diameter of the bone so that there is overhang for potential bony hypertrophy at the bone–implant interface. Antirotation pins were not routinely used, and a preference for 800 pounds per square inch was given whenever there was a sufficient amount of remaining cortical bone. The spindle surface type (hydroxyapatite or porous titanium) was determined by the availability of the implants. Following surgery, all patients were instructed to follow a strict touchdown weight-bearing protocol for 6 weeks, followed by progression to weight-bearing as tolerated.

**Figure 2.**
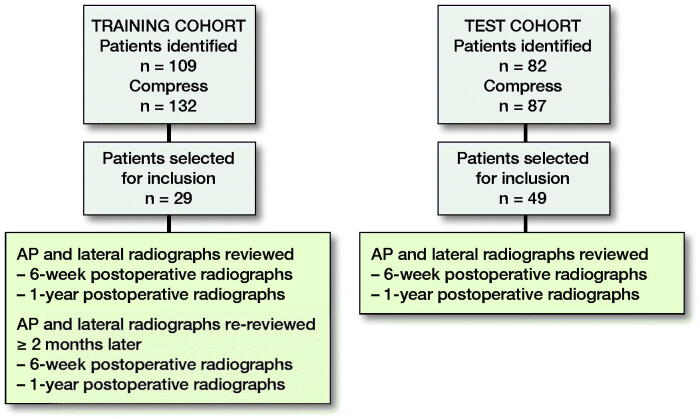
Training and test cohort protocol.

To identify reliable parameters consistent with aseptic mechanical failure, radiographs of 29 patients from this cohort were evaluated (Figure 2). 3 of these patients were known loosenings who went on to revision of the implant. 8 reviewers were asked to assess the 29 sets of patient postoperative radiographs. Each set included anterior-posterior and lateral radiographs taken within 6 weeks post-operation and taken at approximately 1-year postoperatively. Each set of patient radiographs was assessed twice by all reviewers, with the second assessment done no less than 2 months after the first was completed. The reviewers had varying levels of medical education and included 2 orthopedic oncology attending faculty (JBH, YCD), 1 orthopedic resident, 2 musculoskeletal radiology attending faculty, and 3 medical students. In both phases of the study reviewers were blinded to patient name, demographics, and outcomes of the implants. All radiographs were reviewed and scoring was done on our institutional digital PACS system (Agfa Healthcare IMPAX Version 6.5.5.3020; Agfa Healthcare, Mortsel, Belgium). For a power analysis with 8 raters, and an expected ICC of at least 0.7, selecting 29 subjects provided 90% confidence that the ICC would fall within 0.3 of the reported value (Saito et al. 2006).

**Figure 3.**
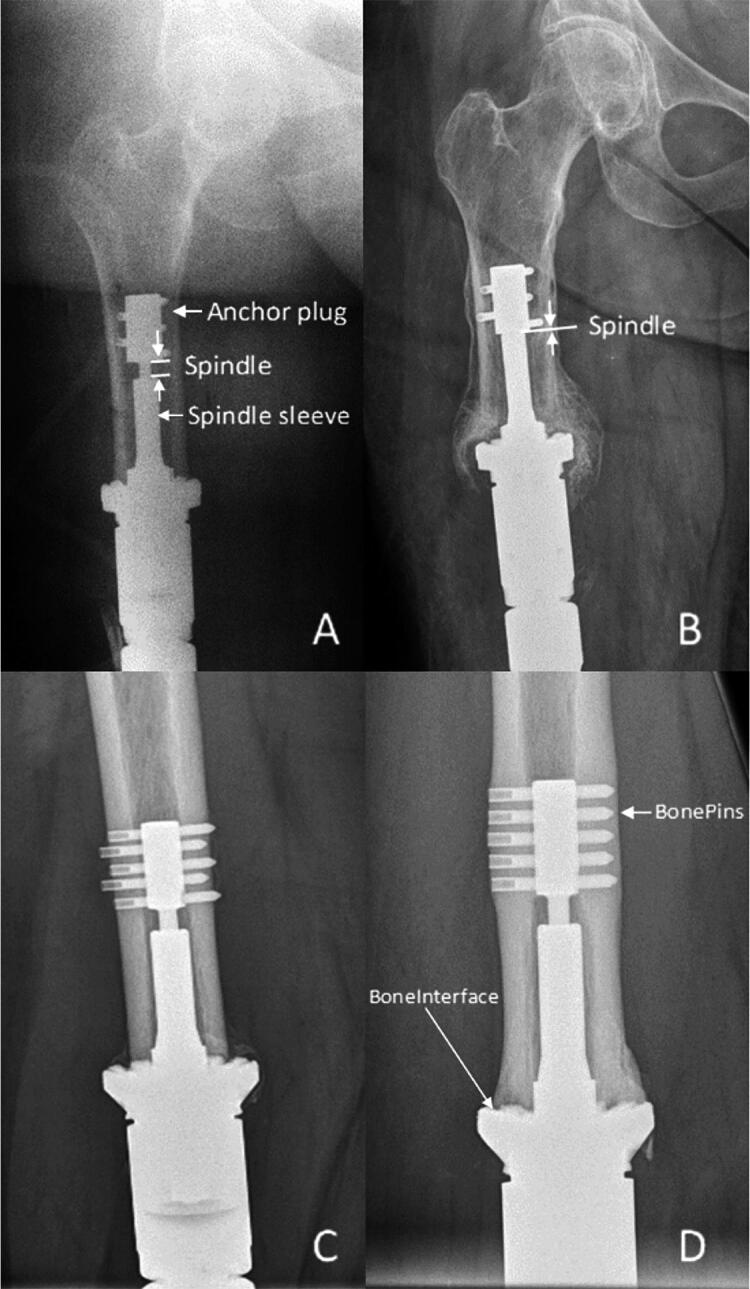
Radiographic parameters used to construct the fast and frugal tree model.

The radiographic parameters assessed were varus/valgus alignment (Coronal), flexion/extension alignment (FlexEx), evidence of bone hypertrophy at the implant (BoneInterface) and at the pins (BonePins), evidence of bone osteolysis (OsteolysisInterface), evidence of intramedullary remodeling (IMRemodel), number of cortices with bone hypertrophy (BoneCortices), difference in bone width (DeltaBone), and distance between the top of the spindle sleeve and the bottom of the anchor plug (Spindle) (Figure 3). In addition, scores similar to RUST and modified RUST were assessed. Scores for each of the 4 cortices were assigned 1 (no change), 2 (hypertrophy with osteolysis), or 3 (hypertrophy without osteolysis). Each cortical score was summed; thus, total scores of bone hypertrophy ranged from 4 to 12 per radiograph.

**Figure 4.**
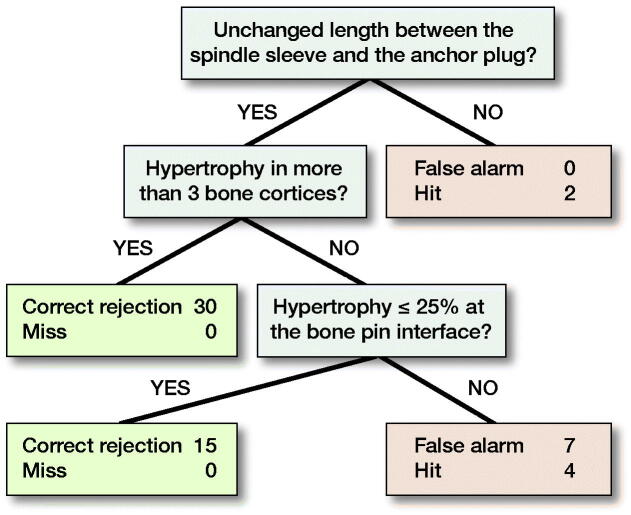
Training cohort FFT.

After radiographs were scored, intraclass correlation coefficients (ICCs) were calculated to assess test–retest and inter-rater reliability. The ICCs were used to determine which parameters to use in constructing the FFT. The final parameters used were: (1) Spindle; (2) BoneCortices; and (3) BonePins (Figure 4). The FFT model was trained to predict aseptic mechanical failure based on the 58 radiographs (2 from each of 29 patients) from this training cohort.

### Test cohort

It is important to test such models separately, to avoid overfitting. Therefore, in addition to the training cohort, we used a second separate cohort to test the FFT. The test cohort consisted of patients who received lower extremity Compress implants between March 2013 and November 2017. During this time surgeons at one center treated 82 patients with 87 Compress implants for lower extremity reconstructions. Indications, inclusion/exclusion criteria, follow-up, surgical specifications, implant location, and postoperative protocol remained unchanged from the training cohort.

**Figure 5.**
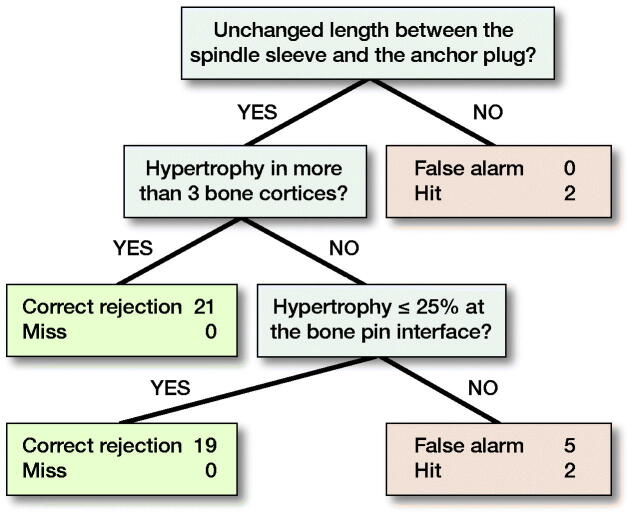
Test cohort FFT. A. Immediate postoperative radiograph of distal femoral reconstruction showing the initial length between the top of the spindle sleeve and bottom of anchor plug (Spindle). B. 1-year postoperative radiograph of the same patient as in A, depicting the loss of length of the Spindle. C. Immediate postoperative radiograph of distal femoral reconstruction. D. 1-year postoperative radiograph of the same patient as in Figure 3C, depicting hypertrophy at both the bone–implant interface (BoneInterface) and at the bone–pin interface (BonePins).

From the 82 patients, anterior-posterior and lateral radiographs from 49 patients were randomly chosen to be reviewed. The reviewers included 2 orthopedic oncology attendings (JBH, YCD) and 1 orthopedic arthroplasty attending (RK) from the same institution. They each reviewed 49 patient radiographs a single time. Each set included anterior-posterior and lateral radiographs taken within 6 weeks postoperatively and taken at approximately 1-year postoperatively (Figure 2). Each of the three reviewers evaluated the radiographs using the three parameters identified in phase I of the study: (1) Spindle; (2) BoneCortices; (3) BonePins. The scored radiographs were evaluated by the FFT model (Figure 5). Sensitivity, specificity, and balanced accuracy (BACC) (Phillips et al. 2017) were calculated. Statistical analyses were completed in R version 3.5.0 (R Core Team, 2018; R Foundation for Statistical Computing, Vienna, Austria) within RStudio version 1.1.453, including the FFTree package (Phillips et al. 2017).

### Ethics, funding, and potential conflicts of interest

The Oregon Health & Science University institutional review board approved this study and waived requirement for written informed consent. There was no external funding provided for this study. JBH receives royalties from Zimmer Biomet for a product unrelated to this study. No other authors report a conflict of interest.

## Results

When comparing the test–retest reliability of individual reviewers, the intraclass correlation (ICC) p-value was not always significant. 6 of 8 reviewers had an intra-rater ICC that was not statistically significant for 1 or more parameters (Table 1). 6 of 11 parameters had 1 or more reviewers with non-statistically significant intra-rater reliability. Coronal, FlexEx, and MSBH were parameters where 2 or more reviewers had non-statistically significant intra-rater reliabilities (Table 1). Some parameters are less reproducible by raters, and thus would not be useful if incorporated into a clinical model for predicting compressive osseointegration fixation aseptic mechanical failure.

**Table 1.** Training cohort: test–retest intra-rater reliability

	Tester 1		Tester 2		Tester 3		Tester 4		Tester 5		Tester 6		Tester 7		Tester 8	
Parameter^a^	ICC	p-value	ICC	p-value	ICC	p-value	ICC	p-value	ICC	p-value	ICC	p-value	ICC	p-value	ICC	p-value
Spindle	0.98	< 0.001	0.89	< 0.001	0.93	< 0.001	0.93	< 0.001	0.92	< 0.001	0.82	< 0.001	0.86	< 0.001	0.45	0.006
Coronal	0.33	0.04	0.25	0.09	0.87	< 0.001	0.74	< 0.001	0.35	0.03	0.03	0.5	0.22	0.1	0.17	0.2
FlexEx	0.36	0.03	0.62	< 0.001	0.91	< 0.001	0.88	< 0.001	0.36	0.02	0.29	0.06	0.22	0.1	0.67	< 0.001
BoneInterface	0.55	0.001	0.52	0.001	0.82	< 0.001	0.48	< 0.001	0.49	0.003	0.14	0.2	0.69	< 0.001	0.42	0.01
OsteolysisInterface	0.31	0.05	0.48	0.003	0.69	< 0.001	0.99	< 0.001	0.31	0.05	0.77	< 0.001	0.99	< 0.001	0.32	0.04
BonePins	0.59	< 0.001	0.78	< 0.001	0.59	< 0.001	0.79	< 0.001	0.67	< 0.001	0.69	< 0.001	0.81	< 0.001	0.65	< 0.001
IMRemodel	0.24	0.10	0.41	0.01	0.57	< 0.001	0.57	< 0.001	0.39	0.02	0.40	0.01	0.61	< 0.001	0.71	< 0.001
BoneCortices	0.86	< 0.001	0.89	< 0.001	0.92	< 0.001	0.86	< 0.001	0.68	< 0.001	0.73	< 0.001	0.63	< 0.001	0.73	< 0.001
SBH	0.74	< 0.001	0.9	< 0.001	0.92	< 0.001	0.92	< 0.001	0.30	0.06	0.64	< 0.001	0.47	0.004	0.54	0.001
MSBH	0.79	< 0.001	0.89	< 0.001	0.94	< 0.001	0.92	< 0.001	0.28	0.07	0.19	0.2	0.47	0.004	0.42	0.01
DeltaBone	0.87	< 0.001	0.85	< 0.001	0.99	< 0.001	0.54	< 0.001	0.86	< 0.001	0.88	< 0.001	0.75	< 0.001	0.52	0.002

a Spindle: distance between the top of the spindle sleeve and the bottom of anchor plug; Coronal: varus/valgus alignment; FlexEx: flexion/extension alignment; BoneInterface: evidence of bone hypertrophy at implant interface; OsteolysisInterface: evidence of bone osteolysis; BonePins: evidence of bone hypertrophy at anchor plug pins; IMRemodel: evidence of intramedullary remodeling; BoneCortices: number of cortices with bone hypertrophy; SBH: score of bone hypertrophy; MSBH: modified score of bone hypertrophy; DeltaBone: difference in bone width.

While individual reviewers may have had non-statistically significant intra-rater ICCs for 1 or more parameters, all of the inter-rater ICCs were highly reliable (p < 0.001) (Table 2). Both the intra-rater and inter-rater reliability were highest for change in distance between the top of the spindle sleeve and the bottom of the anchor plug (Spindle, ICC = 0.98; ICC = 0.81) and bone cortices with hypertrophy (BoneCortices, ICC = 0.86; ICC = 0.68). Since these parameters had the highest intra- and inter-rater ICC and were statistically significant, they were used as decision points in the FFT (Figure 3). A sensitivity analysis was completed while only including staff orthopedic surgeons and radiologists, without any substantial differences in the results.

**Table 2.** Training cohort: inter-rater reliability

Parameter **^a^**	ICC	p-value
Spindle	0.81	< 0.001
Coronal	0.34	< 0.001
FlexEx	0.46	< 0.001
BoneInterface	0.54	< 0.001
OsteolysisInterface	0.53	< 0.001
BonePins	0.65	< 0.001
IMRemodel	0.32	< 0.001
BoneCortices	0.68	< 0.001
SBH	0.58	< 0.001
MSBH	0.52	< 0.001
DeltaBone	0.76	< 0.001

a See footnote Table 1.

When the radiograph scores were interpreted by the FFT they went through a series of 3 yes or no questions to predict whether aseptic mechanical failure had occurred or not. FFTs are designed with balance in mind. In order to have a balanced FFT there must be a balanced number of nodes representing negative and positive outcome, with the final node being a neutral outcome. The negative and positive outcome parameters elected were Spindle and BoneCortices, with BonePins as the neutral node.

Any time a data point (radiograph) reached a node and the finding was present, a decision was made and the data point exited the FFT. Those data points that did not exit continued to the next node on the FFT. Predicted failures exited on the right side of the table while those not predicted to fail exited to the left. Of the 58 radiographs in the training cohort, 2 demonstrated change in the Spindle parameter and thus exited to the right. Of the remaining 56 radiographs that continued to the next question, 30 were found to have hypertrophy in more than 3 bone cortices (BoneCortices) and exited to the left. The 26 remaining data points continued to the final neutral question, where 15 exited left because they had no pin hypertrophy and 11 exited right due to pin hypertrophy; of these, 7 were false positives and 4 were true failures (Figure 3). The predictions made by the FFT were then compared with the clinical outcomes associated with each patient. Of the 13 radiographs that were predicted to fail, 6 were true failures. All of the radiographs that were predicted not to fail did not end up failing. Thus, the sensitivity of the FFT was 100% (95% CI 52–100) and the specificity 87% (CI 74–94), BACC 93.

In the training cohort, failure was always indicated when there was an observable change in Spindle parameter although this measurement alone was not sensitive. Furthermore, whenever there was hypertrophy visible at the bone–implant interface, failure did not occur. Finally, while hypertrophy at the bone pins was predictive of failure, failure did not always result when this was observed on the radiograph.

The same FFT created for the training cohort was used to evaluate the test cohort. 49 radiographs, each scored by 3 reviewers, were run through the FFT with similar outcomes to the training cohort. 2 radiographs demonstrated changes in the Spindle parameter and were predicted to fail, so they exited right. 47 radiographs continued to the second question, and of those 21 demonstrated hypertrophy in 3 or more bone cortices and thus were predicted not to fail and exited to the left. 26 radiographs reached the final question before exiting the FFT. 19 of those did not demonstrate hypertrophy at the bone pins and thus exited to the left and were not expected to fail. Meanwhile, 7 displayed bone pin hypertrophy and exited to the right, because they were expected to fail. In comparison with clinical outcomes, of the 9 radiographs that were expected to fail, only 4 did. Additionally, all of 40 radiographs anticipated not to fail, did not fail. Similar to the training cohort, the FFT for the test cohort was determined to be 100% (CI 40–100) sensitive and 89% (CI 75–96) specific, BACC 94 (Figure 5).

The model was then applied to the subset of patients with distal femoral reconstructions. We identified 24 distal femoral reconstructions, which included 4 failures. The FFT had a 100% sensitivity with 3 false positives, for an 85% specificity.

## Discussion

Compressive osseointegration is an alternative to intramedullary fixation for endoprosthetic reconstruction, with modes of failure that present differently on radiographs. We found that the test–retest reliability of individual reviewers was not always statistically significant, illustrating that some parameters are less reproducible by raters. While individual reviewers may have had non-statistically significant intra-rater ICCs for 1 or more parameters, all of the inter-rater ICCs were highly reliable (p < 0.001). The intra-rater and inter-rater reliability were highest for the Spindle parameter (ICC 0.98; ICC 0.81) and Bone Cortices with Hypertrophy (ICC 0.86; ICC 0.68) and these parameters were used as decision points in the FFT. The sensitivity and specificity of the FFT was 100% and 87%, respectively, for the training cohort and 100% and 89%, respectively, for the test cohort. This model may be helpful for ruling in, and even more helpful for ruling out, loosening.

We found the test–retest reliability of individual reviewers’ p-value was not always significant. Given that the reviewers had different levels of training, it is reasonable to suspect that reviewers with the least training were less capable of reproducing the same results when reading the same radiograph for a second time. It is possible that reviewers 5, 6, and 7 had the least training given that reviewer 6 had non-significant intra-rater ICCs for 4 parameters, and reviewer 5 and 7 each had non-significant intra-rater ICCs for 2. In the development of the RUST score the authors (Whelan et al. 2010) also included reviewers with varying levels of education, from trainee (resident) physicians, to community orthopedic surgeons and fellowship-trained surgeons. They found a trend towards improved reliability for the fellowship-trained traumatologists compared with those with less training.

While the individual reviewers may have had non-statistically significant intra-rater ICCs for 1 or more parameters, all inter-rater ICCs were highly reliable suggesting that once individuals become proficient at reading radiographs, they will be able to reproduce their findings. It is likely that statistical significance was achieved for all 11 inter-rater ICC parameters since 6 of the 8 reviewers were either faculty level or in their postgraduate medical education.

Pedtke et al. (2012) suggested 5 separate radiographic parameters suggestive of aseptic failure of osseointegration that were evaluated by the treating surgeons; however, interobserver variability was not assessed. 1 of the suggested parameters was a progressive gross decrease in the distance between the anchor plug base and the top of the spindle sleeve. We found that both the intra-rater and inter-rater reliability were high for this radiographic parameter we called “Spindle.” Pedtke et al. along with multiple other authors (Kramer et al. 2008, Healey et al. 2013, Zimel et al. 2016) have also suggested that bone hypertrophy at the bone–implant interface, what we called “Bone Cortices with Hypertrophy,” was suggestive of stable osseointegration. We also found this to have high intra-rater and inter-rater reliability. Since these parameters had the highest intra- and inter-rater ICCs and were statistically significant, they were used as decision points as we developed our decision tree.

Our model indicates that if the spindle is stable (has not changed height), and at least 3 cortices have bone hypertrophy, then stable osseointegration has occurred. However, if the spindle is stable, but there are less than 3 cortices with hypertrophy, then hypertrophy around the pins can be considered. Based on our model, bone pin hypertrophy was predictive of failure but failure did not always result when this was present on radiographs. It is possible that this may only be a clue as to inadequate or delayed bone healing at the implant–bone interface.

Our study has a number of limitations. First, there currently is no gold standard with which to compare our decision tree. However, our results support the use of the FFT to create a standardized protocol for future investigations of compressive osseointegration and we hope our analysis is verified or modified in the future. Second, we found 100% sensitivity but only 87% specificity in the training cohort, and 89% specificity in the test cohort. This suggests that this decision tree is likely more helpful as a supplemental tool that clinicians may use for ruling out loosening. Third, we did not have patient-reported outcomes or pain scores to correlate with the decision tree; these findings may also be associated with loosening and future investigations may consider including these clinical findings. Fourth, our training cohort was only 29 patients and there can be limitations due to this relatively small sample size. The study was powered to evaluate inter-rater reliability in the training cohort. While a strength of the study is the separate testing cohort, ultimately the sample size is limited by the relative infrequency of the scenarios for which these implants are used. Finally, this is 1 center’s experience, with surgeons and radiologists who have experience evaluating compressive osseointegration fixation. The results may not be generalizable to other centers, and the external validity of this model would benefit from evaluation in additional cohorts.

This study takes a combination of radiographic parameters in a systematic approach to create a decision tree that may be utilized by clinicians evaluating compressive osseointegration fixation. Our decision tree showed high sensitivity and slightly lower specificity suggesting that this model may help clinicians rule out aseptic mechanical failures. Future studies should be performed to potentially improve on our decision tree by utilizing clinical outcomes or advanced cross-sectional imaging studies. Additionally, as there is currently no gold standard to evaluate compressive osseointegration, we hope this is simply a first step and a supplemental tool for clinicians and is improved upon in the future.
